# Food, microbes, sex and old age: on the plasticity of gastrointestinal innervation

**DOI:** 10.1016/j.conb.2019.12.004

**Published:** 2020-06

**Authors:** Tomotsune Ameku, Hannah Beckwith, Laura Blackie, Irene Miguel-Aliaga

**Affiliations:** 1 MRC London Institute of Medical Sciences, Imperial College London, Hammersmith Campus, Du Cane Road, London W12 0NN, UK; 2 Faculty of Medicine, Imperial College London, Hammersmith Campus, Du Cane Road, London W12 0NN, UK

## Abstract

The gastrointestinal tract is innervated by its own enteric nervous system and by extrinsic neurons that connect it with the central nervous system. Innervation allows the gastrointestinal tract to sense and respond to diverse stimuli, adjusting motility and secretion, but also affecting our physiology, behaviour and immunity. The mechanisms underlying the formation of gastrointestinal neurons are beginning to be elucidated; those that keep them plastic over an organism’s lifetime remain to be explored. Here, we review the effects of microbiota, nutrients, sex and ageing on the morphology and function of gastrointestinal innervation in mammals, and discuss how this plasticity shapes gut-brain crosstalk and whole-body physiology. We also highlight insights gained by nascent studies of the enteric innervation of *Drosophila melanogaster*.

## Highlights

• The gastrointestinal tract is innervated by the ENS and connected with the CNS.
• The gut-brain axis adjusts gut physiology in response to environmental cues and internal state.
• The anatomy and functions of gastrointestinal neurons can change during an organism’s lifetime.
• This plasticity is modulated by factors such as microbiota, nutrients, sex and age.

## Graphical abstract

**Figure:**
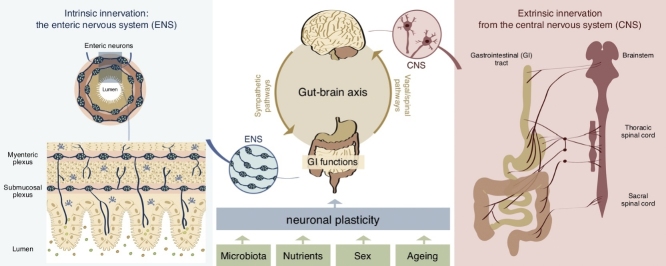

**Current Opinion in Neurobiology** 2020, **62**:83–91

This review comes from a themed issue on **Brain, gut and immune system interactions**

Edited by **Isaac Chiu** and **Asya Rolls**

For a complete overview see the Issue and the Editorial

Available online 3rd February 2020

**https://doi.org/10.1016/j.conb.2019.12.004**

0959-4388/© 2020 The Authors. Published by Elsevier Ltd. This is an open access article under the CC BY license (http://creativecommons.org/licenses/by/4.0/).

## Introduction

**Figure 1:**
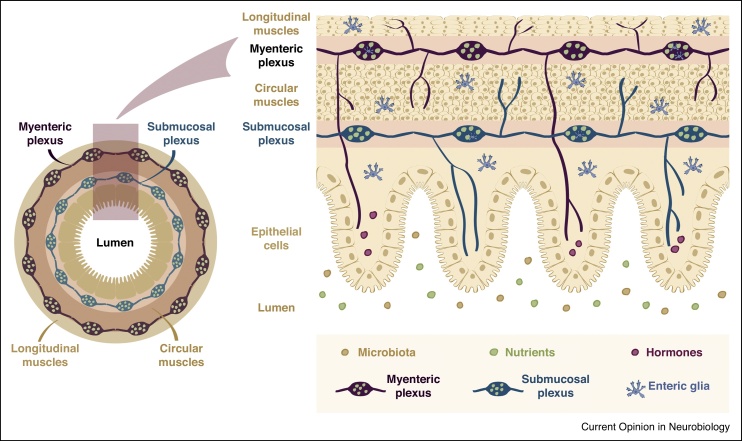
Basic anatomy of the ENS. Schematic representation of a cross section of the GI tract (left) and its tissue layers (right). Epithelial cells are arranged in crypts and villi, which surround the intestinal lumen. These, in turn, are surrounded by two layers of smooth muscles: an inner circular muscle layer and an outer longitudinal muscle layer. The ENS ‘ground plan’ consists of two interconnected enteric plexi: an inner submucosal plexus (in blue) between the epithelial and circular muscle layers, and an outer myenteric plexus (in purple) between the longitudinal and circular muscle layers. Together, they innervate circular and longitudinal muscles, muscularis mucosae, intrinsic arteries and the mucosa. Both plexi consist of different neuronal and glial cell types; 24 neuronal and 3–7 glial subsets have been described in the myenteric plexus, although there are regional differences and the full diversity of mature neuronal and glial types remains to be characterised, especially in the submucosa [1,4^••^,^6^,^7^].

**Figure 2:**
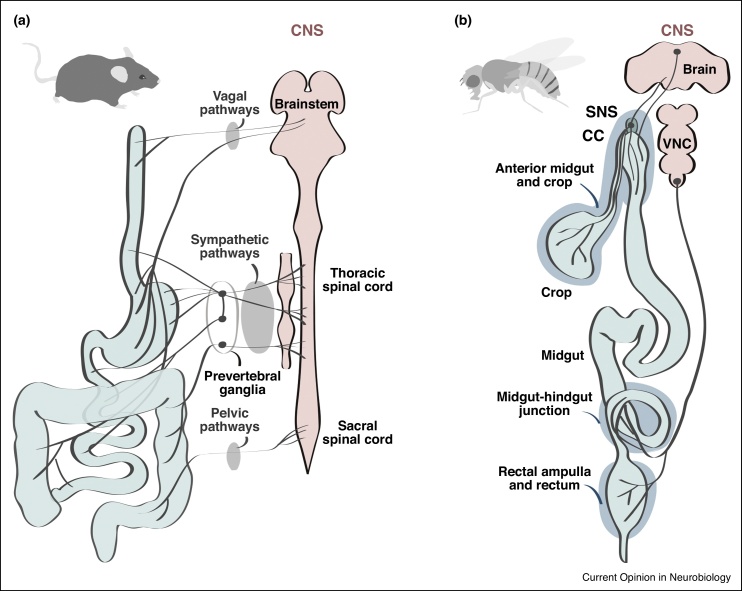
Comparative anatomy of gastrointestinal innervation in adult mice and *Drosophila melanogaster*. Main connections between the central nervous system of adult mice **(a)** and flies **(b)** are depicted. In mice and other mammals, extrinsic innervation through parasympathetic (vagal and pelvic nerves) and sympathetic pathways is apparent throughout the entire length of the GI tract. CNS-gut connections also involve relays via sympathetic ganglia such as prevertebral ganglia. In flies, CNS-gut connections are direct and confined to two main nerves. One nerve emanates from central neurons with cell bodies in the brain’s *pars intercerebralis* and innervates the ENS-like stomatogastric nervous system (SNS) ganglia, the *corpus cardiacum* (a peripheral secretory gland that produces glucagon-like Adipokinetic hormone) and anterior portions of the GI tract (stomach-like crop and anterior portion of the small intestine-like midgut). This nerve is perhaps analogous to the vagus nerve. The other nerve emanates from neurons located in posterior segments of the ventral nerve cord (analogous to the sacral spinal cord) and, like mammalian pelvic/sacral pathways, innervates the posterior midgut, hindgut and rectal ampulla (analogous to ileum, colon and rectum, respectively). CNS-gut communication is bidirectional in both cases, although less is known about sensory pathways in flies. See Refs. [1,50^•^] for details. Abbreviations: CNS, central nervous system; SNS, stomatogastric nervous system; VNC, ventral nerve cord; CC, *corpus cardiacum*.

**Figure 3:**
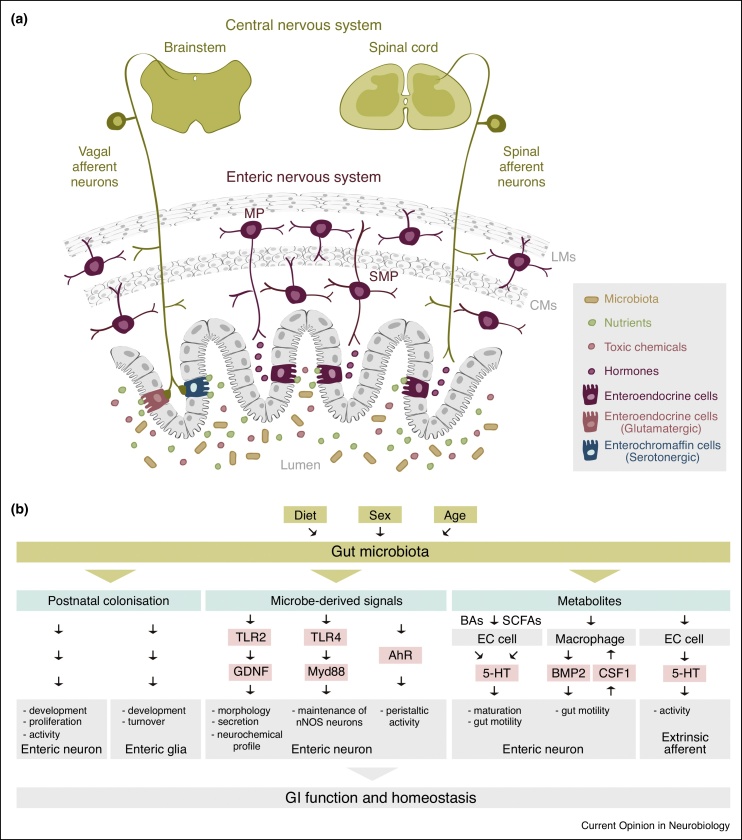
Sensory innervation of the mammalian GI tract. **(a)** The GI tract is innervated by both intrinsic (ENS-borne) and extrinsic sensory neurons. Intrinsic afferent neurons (in dark purple) emanate from the myenteric and submucosal plexi. Extrinsic vagal and spinal afferent neurons (in dark yellow) emanate from the nodose ganglia and dorsal root ganglia, and relay GI stimuli to the brain stem and spinal cord, respectively. Epithelial enteroendocrine cells sense luminal contents. They relay this information via direct synaptic contacts with extrinsic nerves (glutamatergic for cholecystokinin or PYY-positive enteroendocrine cells, serotonergic for enterochromaffin cells) or via enteroendocrine-derived hormones that are sensed by both extrinsic and intrinsic (ENS) afferents. Abbreviations: MP, myenteric plexus; SMP, submucosal plexus; LM, longitudinal muscle; CM, circular muscle. **(b)** Molecular mechanisms involved in the detection of GI microbes by GI sensory neurons. Microbes control the development and function of enteric neurons and glia. Microbe-derived signals are recognised by Toll-like receptors and Aryl hydrocarbon receptor. Microbes also produce metabolites such as secondary bile acids and short-chain fatty acids. Microbe-derived metabolites modulate the activity of enterochromaffin cells, muscularis macrophages, and extrinsic afferents. Abbreviations: TLR, Toll-like receptor; AhR, Aryl hydrocarbon receptor; 5-HT, 5-hydroxytryptamine; BMP, bone morphogenetic protein; BAs, bile acids; SCFAs, short-chain fatty acids; EC, enterochromaffin; GI, gastrointestinal.

Since its discovery almost two centuries ago, the ‘second brain’ of our gastrointestinal (GI) tract has fascinated us by its ability to control our inner workings independent of the actions of our brain. This complex autonomous network is referred to as the enteric nervous system (ENS) and consists of millions of cells, parsed into many anatomical, chemical and functional ENS subclasses including inter, sensory and motor neurons as well as diverse types of glia [1]. These are collectively organised in two layers of interconnected circuits surrounding the GI tract: an outer myenteric plexus for GI motility control, and an inner submucosal plexus for secretion control (Figure 1). The ENS is bidirectionally connected to the CNS; major connections involve vagus and pelvic nerves as well as sympathetic pathways [1] (Figure 2a). Both this extrinsic innervation and the ENS itself are able to sense GI status (nutrients, distension, microbes, hormones) via specialised sensory afferents (Figure 3). Intrinsic ENS afferent neurons reside in both the enteric and submucosal plexi of the ENS, whereas extrinsic vagal and spinal afferent neurons originate from peripheral ganglia and relay GI information back to the CNS [2].

Important mediators of ENS development have been identified, mutations in which have been shown to underlie congenital disorders [3]. Recent work is also shedding light on the diversity of both ENS [4^••^,5^••^,^6^,^7^] and vagal [8^••^] cell types, revealing previously unrecognised morphological and chemical diversity, and beginning to uncover mechanisms that account for their spatial and functional organisation.

Less is known about the mechanisms that keep GI innervation plastic over an organism’s lifetime. Further investigation is timely in light of increasingly recognised roles for the GI tract beyond digestion. Indeed, our GI tract can sense, integrate and react to nutrients, microbiota or internal state to impact our physiology, behaviour and even immunity. It is therefore reasonable to expect its nervous system to contribute to, and be remodelled by, these adaptations. This review summarises recent work on the plasticity of GI innervation — both morphological and functional — and its significance. We focus on the impact of microbes, nutrients, sex and age on mammalian enteric neurons, and highlight insights gained by nascent studies of the enteric innervation of *Drosophila melanogaster*.

## The microbes within: modulation of enteric neurons by microbiota

The existence of a microbiota-gut-brain axis is now widely accepted. Absence of microbiota has been associated with distinct physiological and behavioural changes, and various neurodevelopmental or neurodegenerative disorders are associated with changes in microbiota [9]. To move beyond correlations, recent work has explored how bacteria talk to the host. Might enteric neurons and/or glia play a role in this context?

Although the ENS is anatomically organised during foetal life, interaction with gut bacteria begins postnatally as bacteria begin to colonise the GI tract. The importance of microbiota-ENS interactions was suggested by the abnormalities in morphology and activity of myenteric neurons [10, 11, 12, 13, 14] and a failure of development of enteric glial cells [15] observed in germ-free mice. The latter study further revealed roles for the gut microbiota in sustaining previously unrecognised homeostatic turnover of enteric glial cells in adult life. Re-colonisation of germ-free mice can also induce proliferation of myenteric cells expressing the neuronal precursor marker Nestin [16^•^] and reduce their transit time deficit [14].

What molecular mechanisms mediate microbiota-ENS interactions? Microbe-derived signals are recognised by Toll-like receptors (TLRs): an interaction required for maintaining intestinal epithelial homeostasis [17,18]. The ENS expresses both intracellular TLRs (TLR3 and TLR7), which recognise viral RNA, and plasma membrane-bound TLRs (TLR2 and TLR4), which interact with molecules composing the bacterial cell walls and membranes [19]. Microbially derived signals control ENS plasticity through TLRs; *TLR2* mutant mice show abnormalities in enteric neurons [20], and *TLR4* mutant mice show reduced number of nNOS-inhibitory neurons and delayed gut motility reminiscent of germ-free mice [11]. Besides recognition by TLRs, microbiota can also influence the ENS by contributing to the production of metabolites such as short-chain fatty acids and secondary bile acids, which promote serotonin production from enterochromaffin cells [21]. Serotonin, in turn, controls maturation of the adult ENS via serotonin signalling within enteric neurons [16^•^]. Finally, microbiota also activate the Aryl hydrocarbon receptor (Ahr) transcription factor in colonic ENS neurons, which is not required for ENS neuron development but sustains ENS-dependent peristaltic activity [14]. This study and a few others [16^•^,^22^] are therefore revealing that the actions of microbiota on the ENS may be functional as well as anatomical, impacting GI motility in a diet-dependent way.

Extrinsic afferents may also play a role in microbiota-to-brain communication; they can respond to enteroendocrine cell-derived serotonin following activation of enteroendocrine cells by microbially derived metabolites [23^••^]. Evidence for direct sensing of microbiota by extrinsic afferents is currently limited [24].

It is also becoming apparent that GI neurons engage in extensive crosstalk with macrophages and other immune cells, both in normal homeostasis and in response to infection. The physiological significance of these neuro-immune interactions extends beyond immune functions and is an area of active investigation [25].

Future characterisation of these local interactions between GI neurons and other intestinal cell types — as well as the systemic responses such interactions elicit — will help shed light on how microbes modulate neuronal functions to affect physiology and behaviour (Figure 3b). It will also be of interest to explore contributions of GI neurons to recently described sexual dimorphisms in microbiota and/or their effects on the host [26, 27, 28]. Importantly, we may need to move beyond comparisons involving germ-free mice — somewhat artificial, as animals are rarely germ-free — and consider how different, indigenous microbiota populations shape the anatomy and/or function of enteric neurons and/or glia with physiological consequences. These effects may differ depending on the sex, age and nutritional status of the host (see next sections).

## Food for (gut) thought: modulation of gastrointestinal neurons by nutrients

Intestinal nutrient sensing was historically the role of epithelial enteroendocrine cells; in response to certain nutrients, they would release specific enteroendocrine peptides into the circulation [2]. Enteroendocrine peptides such as incretins (GIP, GLP-1, GLP-2) can also have more local effects on enteric neurons or vagal afferents, which express enteroendocrine peptide receptors [29,30]. Nutrients can, however, be directly sensed by both enteric neurons and extrinsic sensory afferents once they have been absorbed and/or metabolically transformed. An extensive review has described the effects of nutrients on the ENS [31]. We provide a summary of these findings as well as more recent findings pointing to important roles for extrinsic sensory neurons in relaying these nutritional signals.

ENS neurons express and are activated by transporters/receptors for amino acids, glucose and fatty acids [2]. Some nutrients may also affect ENS activity indirectly by modulating the synthesis of specific neurotransmitters. For example, l-cysteine is a substrate for enzymes involved in synthesis of the enteric neurotransmitter, nitric oxide [31]. The functions modulated by ENS neuron nutrient sensing remain to be fully characterised; effects on motility, secretion and/or absorption are plausible.

Whilst the intrinsic innervation of the intestine would appear at first sight to be best placed to receive nutritional signals, given that submucosal ganglia are closer to absorption sites and vessels (Figure 3), it has been known for decades that nutrients can also activate vagal afferents [31]. This may enable rapid transmission of nutritional signals from gut to brain. Recent work is beginning to shed light on the neuronal types involved. For example, two distinct types of vagal sensory neurons monitor or control digestion: GLP1R neurons act as mechanosensors, detecting stretch in the intestinal muscle and stomach, whereas GPR65 neurons innervating the intestine detect nutrients and control gut motility [32]. A recent study has further illustrated the importance of mechanosensory mechanisms within vagal sensory neurons [8^••^]. There is also increasing evidence that nutrient-sensing enteroendocrine cells form direct synaptic connections with extrinsic sensory neurons [23^••^,33^••^]. The net effect of vagal activation was thought to be negative on food reward, but a recent study challenged this view by revealing that vagal afferents can also transmit reward signals to the brain [34^••^]. A more integrated view of how different nutrients are sensed — both at the periphery and internally — is likely to emerge in coming years, which should consider the temporal dynamics of neuronal responses as well as their effects on ingestion dynamics, food reward and preference [35].

As well as relaying nutritional signals, GI neurons can be persistently remodelled by longer-term nutrient scarcity or excess. For example, fasting enhances activation of myenteric neurons induced by the hunger hormone ghrelin but not by the satiety hormones cholecystokinin or serotonin [36]. Additionally, feeding of a resistant starch-supplemented diet, which induces the production of the short-chain fatty acid butyrate, selectively increases the proportion of cholinergic neurons [37]. There is also increasing evidence that persistent exposure to a high-fat diet can result in ENS sensitisation and (somewhat more disputed) loss and/or damage of myenteric neurons and gut dysmotility; both GI dysmotility and enteric neuropathy are also associated with type 2 diabetes in humans [31,38]. Although the mechanisms coupling nutrient availability with its long-term effects on the ENS remain to be fully investigated, hormones are attractive candidates. ENS functions are modulated by intestinal and gastric peptide hormones — such as glucagon-like peptide, cholecystokinin, ghrelin and leptin — as well as neuropeptides — such as neuropeptide Y and galanin [39]. It will be interesting to explore whether specific nutrients remodel the ENS through their actions on specific hormones and *vice versa*: whether hormonal status remodels the ENS to shape the way in which the intestine detects and handles specific nutrients.

## Male or female? Sex differences in gastrointestinal neurons

Many aspects of our physiology are sexually dimorphic and gastrointestinal physiology is no exception. For example, male and female intestines harbour different microbiota, which impact immunity and metabolism in a sexually dimorphic manner [26, 27, 28]. Many GI disorders are more prevalent in females [40]. Are GI neurons the target and/or drivers of these sex differences?

The mammalian GI tract expresses sex hormone receptors for oestrogen, progesterone, luteinising hormone and androgens [40]. Perhaps more surprisingly, it is also a significant source of the sex hormones themselves; intestinal expression of oestrogens and gonadotropin-releasing hormone (GnRH) has been reported [40]. Although their expression in GI neurons has not been systematically investigated, there is some evidence for the presence of both sex hormones and their receptors in enteric nerves: GnRH and luteinising hormone receptor in humans and rats, and oestrogen receptors in humans, rats and mice [40].

Sex hormones may directly affect ENS functions. Treatment with an oestrogen receptor β agonist induced proliferation of enteric glial cells and differentiation of mature neurons after damage [41]. It also protected against loss of myenteric neurons induced by a high-fat diet, or by a Parkinson’s disease model [40,41]. In contrast to these neuroprotective effects of oestrogen, treatment with a GnRH analogue reduced enteric neuron number in rats and humans [42]. Changes in sex hormone levels may also affect GI function in pregnancy: oestrogen — but not progesterone — increased the number of nNOS-inhibitory neurons in the myenteric plexus of the stomach and colon, which may contribute to the pregnancy-associated delay in gastric emptying and colonic transit [43].

Whether the sex of the ENS and/or the extrinsic innervation of the GI tract underlies sex differences in physiology remains to be established. In this context, it may become important to consider cell-intrinsic mechanisms as well as hormones; in the fruit fly, the cell-intrinsic (sex chromosome-determined) sexual identity of intestinal epithelial stem cells makes them divide more frequently in females [44]. This allows the female intestine to increase its size during pregnancy, but also renders it more vulnerable to tumorigenic insults [44]. In both flies and mammalian systems, it will be of interest to explore whether similar cell-intrinsic mechanisms contribute to sex differences in GI neuron functions and/or the sex bias of GI disorders in which the ENS is known to play a role. Intriguingly, the expression of the Y chromosome-encoded sex determinant SRY appears to be upregulated in ENS neurons of male Hirschsprung’s disease patients: a disorder that predominantly affects males [45].

## Guts and old age: impact of ageing on gastrointestinal neurons

Many GI neurons are continually exposed to mechanical stress and chemical insults from luminal contents; the longer an animal lives, the more stimuli/challenges to which they are subjected. Ageing affects GI neuromuscular functions that influence a variety of digestive disorders [46]. A number of studies on different animal models have reported an age-related loss in myenteric neurons in the small and large intestines, although there are some discrepancies regarding the extent of this decline [46]. Until recently, there was little evidence for ongoing neurogenesis in the healthy adult intestine, but the regenerative capacity of the mammalian ENS is now beginning to be recognised and described [16^•^,^47^,48^•^]. Deeper insight into the nature of these adult ENS precursors and their regenerative potential will help clarify potential contributions of enteric neuronal turnover (or lack thereof) to chronic ENS pathologies.

The mechanisms by which ageing affects GI neurodegeneration remain to be elucidated but food calories, oxidative stress, changes in neurotrophic factors, and calcium dysregulation all may contribute [46,47]. An age-related increase in inflammation is also a good candidate for contributing to neuronal loss in the ENS; an age-dependent shift in macrophage phenotype causes inflammation-mediated degeneration of the ENS. This shift is associated with age-related changes in gut microbiota composition, pointing to contributions of the gut-immune-microbiota axis to the age-dependent changes in ENS functions [49].

Understanding the mechanisms underlying the decline of GI innervation during ageing may not only provide an entry point into therapeutic interventions aimed at halting this ‘natural’ decline, but also other conditions associated with enteric neuropathy including diabetes and Parkinson’s disease. Not all age-dependent neuronal changes may be degenerative; some may fulfil adaptive roles to counteract the decline of other GI cell types. Distinguishing between adaptive and detrimental changes will require comprehensive characterisation of how specific neurons age —morphologically, transcriptionally, functionally — and genetic interrogation of the functional significance of such changes.

## Plasticity of gastrointestinal neurons in *Drosophila*: an integrative view from a simpler gut

Elucidating the significance of GI innervation has met with two significant challenges. One is its daunting complexity, which we are only beginning to parse. In order to connect key signals and neurons with the physiological processes they regulate, we also require temporal, spatial and specific control of gene expression and/or neuronal function in defined neuronal subsets. Refined viral approaches, advances in genome engineering and improved methods to manipulate neuronal activity are beginning to identify relevant circuitry in mice. Alternatively, we can leverage available genetic tools in experimental systems that allow such temporal and spatial precision, and combine them with genetic screens to more comprehensively probe physiological contributions of specific neurons and/or genes. *Drosophila melanogaster* is one such system.

The innervation of the adult digestive tract of *Drosophila* is complex yet sparser and more spatially restricted than that of the mouse (Figure 2). In the adult fruit fly, gut-innervating extrinsic neurons reside in the CNS and directly extend their axons toward three distinct regions of the digestive tract. ENS-like ganglia are also found in anterior portions of the intestine (oesophagus and midgut portion analogous to stomach/duodenum). Like in mammals, extrinsic and intrinsic innervation are connected, probably bidirectionally [50^•^].

In adult flies, subsets of GI neurons can be readily activated/silenced, and genes can be downregulated/overexpressed exclusively within them, either acutely or longer-term. The activity of these neurons can then be assessed *in vivo* using calcium imaging, as can their effects on intestinal features (e.g. peristalsis) or whole–body physiology. These approaches have identified neurons that lead to long-term nutrient-dependent remodelling of the fly ‘vasculature’ (an oxygen-delivering tubular network referred to as trachea) during larval growth, which promotes survival in nutrient scarcity [51]. In the adult stage, the gut-brain axis can regulate multiple aspects of fly physiology. For example, central gut-innervating neurons have been identified that control intestinal fluid homeostasis [52], regulate excretion in response to sugars [53] or adjust intestinal physiology during reproduction [52]. Importantly, flies have also uncovered new ways in which the gut and brain communicate; a case in point is the finding that male-biased citrate production by enterocytes promotes food intake via a neuronal relay [54^••^]. In future, *Drosophila* could be used to investigate how GI neurons are modulated by age, nutrition, sex and reproductive status, or to explore the significance of inter-cellular/inter-organ crosstalk — for example, whether and why enteroendocrine cells ‘talk to’ GI neurons.

## Conclusions and outlook

Environmental and internal signals can lead to long-term changes in GI neurons. These can differ between males and females, and as animals age. Both ENS and extrinsic neurons respond to these triggers to drive physiological adaptations, but the links between specific nutrients, microbiota or sex determinants, the neuronal adaptations they elicit and their long-term physiological consequences remain to be established. Investigation of such links will shed light on how the GI tract controls behaviour and immunity, and will increase our understanding of human ENS disorders.

Looking ahead, it will be important to distinguish what aspects of GI plasticity stem from the ENS ability to continue to replenish itself in adult life, from those resulting from changes in already differentiated neurons and glia. Because ENS neurons are highly interconnected and are generated asynchronously, effects on one neuronal population may impact other ENS/extrinsic neurons; a case in point is the effect of serotonin derived from early born neurons on later-born neuronal cell types [55]. Finally, it may be important to consider that GI neuron plasticity may extend to other, less obvious features; might the nervous system of the gut be able to learn and remember? [56].

## Conflict of interest statement

Nothing declared.

## References and recommended reading

Papers of particular interest, published within the period of review, have been highlighted as:

• of special interest
•• of outstanding interest
